# Unraveling the Enigma of Belly Dancer's Dyskinesia: A Detailed Case Analysis in the Context of Schizophrenia

**DOI:** 10.7759/cureus.49796

**Published:** 2023-12-01

**Authors:** Bayan Berri, Yisroel Y Grabie, Mark Tawfik, Svetlana Volovich

**Affiliations:** 1 Department of Internal Medicine, CUNY (City University of New York) School of Medicine, NYC, USA; 2 Department of Internal Medicine, Northwell Health - Staten Island University Hospital, Staten Island, USA

**Keywords:** promethazine, baclofen, antipsychotics, hiccups, abdominal dyskinesia

## Abstract

Belly dancer's dyskinesia (BDD) is an unusual neurological disorder characterized by focal dyskinesia that results in involuntary, rhythmic movements of the anterior abdominal wall. This case report comprehensively examines the presentation, potential medication-induced etiology, and therapeutic response of a 64-year-old male diagnosed with schizophrenia. The patient developed BDD-like symptoms resembling hiccups, experiencing recurrent episodes that endured for hours and occurred nearly daily, significantly affecting wakefulness and sleep. Importantly, the patient's medical history included the utilization of fluphenazine and benztropine for schizophrenia management. Following a thorough multidisciplinary neurology consultation, a tailored treatment regimen involving clonazepam, promethazine, and baclofen was initiated, culminating in a noteworthy reduction in symptom frequency. This report substantially enriches the existing knowledge of BDD, highlighting the critical necessity to elucidate its intricate pathophysiology for the advancement of refined diagnostic and therapeutic strategies.

## Introduction

Belly dancer's dyskinesia (BDD), as described by Gupta and Kushwala in 2017, represents a rare neurological disorder characterized by focal dyskinesia afflicting the anterior abdominal wall [1]. Although several instances have been meticulously documented, the precise mechanistic underpinnings remain elusive. As discussed in Rathmann et al.'s 2022 paper, this disorder may manifest from diverse causative factors, including pharmacological agents, neurodegenerative cerebral disorders, spinal neoplasms, and even pregnancy [2]. Of notable significance is the mediating role of pharmaceutical agents in precipitating BDD, with substances such as L-Dopa, quetiapine, prochlorperazine, domperidone, clebopride, and salbutamol implicated in this context, as explained by Rathmann et al.'s 2022 research [2]. Gupta and Kushwala’s study notes how the customary clinical presentation typically encompasses painless rhythmic movements of the anterior abdominal wall, occasionally waning during periods of slumber [1]. Rathmann et al.’s report outlines noteworthy strategies for symptomatic management, including benzodiazepines, sodium channel modulators, botulinum toxin infiltrations, transcutaneous electrical nerve stimulation, and deep brain stimulation [2].

## Case presentation

We present a case of a 64-year-old African American male with a past medical history of type II diabetes mellitus, hypertension, and schizophrenia managed by utilizing fluphenazine and benztropine. He was on benztropine due to the development of extrapyramidal symptoms secondary to fluphenazine. He presented with unrelenting hiccups, yet on detailed physical examination, conspicuous rhythmic involuntary contractions of the abdominal region manifested at intervals of 5 to 10 seconds. The contractions were predominantly localized to the abdominal midline. Although the patient disavowed any sensations of pain, cough, or breathlessness, he did acknowledge sporadic substernal burning sensations, notably in the supine position. Following treatment with gabapentin, the symptoms endured unabated. The patient reported an abrupt onset of symptoms approximately three weeks prior during a dinner meal, persisting for several hours daily, inducing discomfort without associated pain. Notably, the symptoms manifested during both wakefulness and sleep, consequently disrupting the patient's sleep patterns. Subsequent neurological consultation led to the prescription of promethazine and clonazepam, inducing cessation of the BDD episode. While magnetic resonance imaging (MRI) investigations of the thoracic and lumbar spine proved inconclusive due to the patient's limited tolerance, imaging of the brain, cervical spine, and magnetic resonance angiography (MRA) of the cranium and neck revealed no remarkable findings. Subsequent administration of baclofen three times daily and as-needed promethazine resulted in a marked reduction in BDD frequency, transforming it from occurring for extended durations almost daily to a few instances per week, although the duration of each episode remained unaltered.

## Discussion

Literature review 

The existing literature underscores the sporadic occurrence of BDD cases, which engenders challenges in unraveling its underlying pathophysiology. While the clinical manifestations have been meticulously described, the mechanisms governing the onset and persistence of this disorder remain elusive. This lacuna is evident in Gupta and Kushwaha's study, which underscores BDD's rarity via their case report while emphasizing the paucity of insight into its causative factors and optimal therapeutic strategies [1]. Rathmann et al.'s research contributes insights into BDD by detailing three additional cases, thereby reinforcing the peculiar nature of the disorder and its potential association with medications and central nervous system dysregulation [2]. The case report by Tafesse Mengesha et al., in 2023, extends the spectrum of BDD triggers, delineating a pregnant patient and raising intriguing prospects concerning spinal cord compression and hemodynamic perturbations [3]. Furthermore, Iliceto et al.'s 1990 report outlines three cases of post-surgical BDD onset, implying surgical interventions as potential etiological contributors to BDD [4].

The intricate pathophysiology of BDD, with its emphasis on potential inhibitory spinal interneuron dysfunction or local neuronal circuit reorganization, has been posited by various authors, including Iliceto et al.’s study [4]. This perspective finds support in clinical observations indicating bilateral BDD occurrences during sleep, suggestive of a central origin. However, instances contradicting this trend also exist, where BDD subsides during sleep, suggesting peripheral, spinal, or psychogenic origins. Imaging modalities, such as MRI, have been harnessed to glean insights into the anatomical basis of BDD. Regrettably, in the present case, the patient's intolerance to MRI procedures curtailed the definitive conclusions attainable from thoracic and lumbar spine imaging.

While BDD remains principally a clinical diagnosis, diagnostic tools, such as comprehensive history-taking, meticulous physical examination, myelography, electroencephalography (EEG), cerebral computed tomography (CT), thyroid function assessment, and cerebrospinal fluid (CSF) analysis, have been advocated by Iliceto et al.'s report [4]. Although these assessments tend to yield normal results in BDD patients, they facilitate a nuanced understanding of factors precipitating, alleviating, or exacerbating BDD symptoms [4]. Noteworthy are instances such as those presented by Iliceto et al.’s case review, where deep inspiration and breath-holding quelled or attenuated abdominal movements, as well as a case where hyperthyroidism was identified on thyroid function tests, and symptom resolution followed hyperthyroidism treatment [4]. Rathmann et al.’s research presented three BDD cases with concurrently low vitamin B12 levels; these patients exhibited improved symptoms following clonazepam and vitamin B12 supplementation [2]. Despite BDD's primarily clinical diagnosis, a comprehensive investigative approach may unveil contributory factors that either mitigate or exacerbate symptoms, thereby facilitating personalized therapeutic interventions.

The heterogeneous etiology of BDD complicates treatment paradigms, as the approach is contingent on the underlying cause. Several cases indicate that discontinuation of the medications implicated in BDD leads to symptom resolution. In 2023, Kaga et al. reported a case where cessation of droxidopa and amantadine entirely terminated abdominal movements [5]. Similarly, Wong et al.’s 2021 report documented symptom amelioration following cessation of salbutamol [6]. In 2021, Ossella et al. detailed a successful case of BDD treatment with oral diazepam in a 14-year-old female patient [7]. However, variable responses are witnessed, as exemplified by Iliceto et al.’s case review, which observed symptom alleviation with trihexyphenidyl treatment in one patient while another remained refractory [4]. The case report by Gupta and Kushwaha discussed clonazepam and promethazine as completely stopping the symptoms of BDD, and they never recurred [1]. Similarly, in the present case, a therapeutic protocol involving clonazepam, promethazine, and baclofen instigated a notable reduction in symptom frequency. Hence, the diversity in BDD's pathophysiology precludes definitive treatment strategies.

Discussion on BDD

BDD is a unique neurological disorder that is characterized by focal dyskinesia of the anterior abdominal wall. BDD remains an exceptionally rare disorder with elusive mechanistic underpinnings, making its diagnosis and treatment a challenge. The intricate interplay of potential causative factors, including pharmacological agents, neurodegenerative processes, and spinal anomalies, demands a multidisciplinary approach to its evaluation.

The conjectural involvement of medications, particularly antipsychotics, in BDD's genesis, remains an ongoing area of scrutiny. In congruence with this notion, the patient in this report exhibited prior extrapyramidal manifestations necessitating the administration of benztropine in conjunction with fluphenazine. The plausible association of fluphenazine with BDD onset resonates with instances where antipsychotics, such as prochlorperazine, have been implicated. This underscores the necessity for judicious consideration of medication-induced BDD, especially in individuals with a history of extrapyramidal manifestations. 

Given these findings, there are some limitations in this case report. For one, the patient had poor memory regarding the onset of his first episode of BDD, making it difficult to get a thorough history. Additionally, he refused to elaborate upon being questioned regarding his recurrent episodes of BDD. He was also unable to tolerate the MRI, which would have provided additional insight into the etiology of his BDD. Furthermore, due to his inability to discontinue fluphenazine and benztropine, it is uncertain if these drugs caused this patient's BDD.

Moreover, as shown in the review of the literature, treatment options for BDD are diverse and individualized, but the challenge of achieving complete symptom resolution persists. The patient's response to a tailored regimen comprising clonazepam, promethazine, and baclofen demonstrates the variable nature of therapeutic outcomes. This patient’s current episode of BDD completely stopped with a single dose of clonazepam and promethazine. The patient was prescribed promethazine and baclofen to modulate the frequency and duration of these symptoms. This emphasizes the need for continued research to elucidate the optimal treatment approaches for BDD, considering its heterogeneous etiology.

## Conclusions

In conclusion, the case of BDD presented here serves as a significant addition to the existing knowledge surrounding this enigmatic neurological disorder. Through a comprehensive exploration of the patient's history, clinical presentation, diagnostic workup, and therapeutic interventions, this report underscores several key take-home points that enhance our understanding of BDD and its management. Ultimately, this case report emphasizes the imperative to unravel the intricate pathophysiology of BDD for the advancement of refined diagnostic tools and effective therapeutic strategies. The rarity and complexity of this disorder necessitate ongoing collaborative efforts among neurologists, psychiatrists, and other healthcare professionals to enhance our comprehension of BDD and improve the quality of life for affected individuals.
